# Tomato-derived carbon quantum dots: composition-driven structure and copper sensing potential

**DOI:** 10.1038/s41598-026-58128-3

**Published:** 2026-06-16

**Authors:** Jovana Periša, Jan Hočevar, Bojana Milićević, Jernej Iskra, Boštjan Genorio, Darja Lisjak, Miroslav D. Dramićanin, Jelena Papan Djaniš

**Affiliations:** 1 Centre of Excellence for Photoconversion, Vinča Institute of Nuclear Sciences, National Institute of the Republic of Serbia, University of Belgrade, Belgrade, 11351 Serbia; 2 Faculty of Chemistry and Chemical Technology, University of Ljubljana, Ljubljana, 1000 Slovenia; 3 Department for the Synthesis of Materials, Jožef Stefan Institute, Ljubljana, 1000 Slovenia

**Keywords:** Tomato biomass, Luminescence, Carbon quantum dots, Chemistry, Materials science, Nanoscience and technology

## Abstract

Carbon quantum dots (CQDs) were synthesized via a green, bottom-up approach from tomato biomass, using stems and fruits as distinct carbon precursors. To tailor their surface chemistry and optical response, m-aminobenzoic acid was used as a nitrogen-containing dopant during synthesis. The resulting CQDs form colloidally stable, nanoscale aggregates in aqueous media and exhibit strong excitation-dependent photoluminescence, with maximum emission under excitation at 300 and 370 nm. Comprehensive structural and surface analyses reveal that precursor origin significantly influences defect density, hybridization states, and functional group distribution, directly governing luminescent behavior. Both fruit-derived and stem-derived CQDs display efficient fluorescence quenching upon coordination with Cu²⁺ ions, enabling sensitive detection with limits of detection of 0.059 µM and 0.048 µM, respectively. The enhanced sensing performance is attributed to surface-accessible oxygen- and nitrogen-containing functional groups that promote strong metal–ligand interactions. This work demonstrates a sustainable and low-cost strategy for converting agricultural waste into functional nanomaterials, while highlighting the critical role of biomass composition and surface chemistry in tuning CQD photophysics and metal-ion sensing capabilities.

**Supplementary Information:**

The online version contains supplementary material available at https://doi.org/10.1038/s41598-026-58128-3.

## Introduction

Agriculture is the main source of biomass in Europe, accounting for approximately 70% of the total biomass available for processing or consumption. Each year, roughly 924 million tonnes of agricultural biomass are produced, with 54% being primary products (such as grains, fruits, roots, and tubers) and around 46% being residues (leaves, stems, straw)^1,2^. After grapes, tomatoes are among the most widely grown vegetables and fruits in the EU, with an annual production of 17 million tonnes. The yearly biomass from tomatoes (unsold fruits, leaves, and stalks) can amount to 3.1 million tonnes^2^. To date, various models exist for valorizing tomato residues, including extracting pigments (e.g., lycopene and carotene)^3,4^ and phenolic compounds^5^, anaerobic digestion to produce biogas^6^, pyrolysis for making biochar^7^ and bio-oil^8^, and composting^9^.

Biomass acts as a versatile source for deriving a wide range of functional materials, supporting applications in sustainable energy, pollution control, biomedicine, and advanced materials, etc^10–19^.

However, turning tomato biomass into sustainable materials remains challenging. One potential use of tomato residues is their conversion into luminescent materials, particularly carbon quantum dots (CQDs). CQDs are low-dimensional carbon nanostructures with adjustable fluorescence features, making them useful in biomedicine, optoelectronics, sensors, and catalysis^20^. CQDs made from natural sources like biomass are eco-friendly and cost-effective. So far, many biomass sources have been used for their synthesis, from forestry residues to agricultural waste^21^. Specifically, CQDs derived from tomato residues have been produced using hydrothermal and microwave methods, resulting in materials with potential for bioimaging of plant pathogenic fungi and for detecting vanillin and herbicide trifluralin^22,23^.

Metal ions, including copper (II), are common environmental pollutants due to their release into water bodies through industrial processes such as mining, metal plating, and the use of copper-based pesticides. Although copper is an essential trace element for living organisms, elevated concentrations of Cu²⁺ can be toxic, as they tend to accumulate in sediments, posing long-term risks to ecosystems^24–26^. In humans, high levels of copper exposure can cause gastrointestinal distress and, in extreme cases, liver, brain, and kidney damage^26,27^. Due to these hazards, regulatory limits are enforced on copper concentrations in drinking water and industrial effluents. Effective removal and monitoring strategies are crucial to reduce its environmental and health impacts. One promising approach to addressing copper pollution involves using carbon quantum dots (CQDs) for sensitive detection and potential removal of Cu²⁺ from contaminated environments^28–34^.

In this paper, we describe a two-step, environmentally friendly hydrothermal synthesis of sustainable CQDs derived from tomato biomass. These CQDs emit vibrant blue/green light and have potential use in detecting Cu^2+^ ions. Two types of CQDs were produced: one from dried fruit (CQD F) and another from the stem (CQD S). The produced CQD F showed its strongest emission at 410 nm when excited at 300 nm, while CQD S had a peak at 450 nm upon 370 nm excitation. Both CQDs also demonstrated tunable fluorescence, with emission wavelengths changing based on the excitation wavelength. The structure, morphology, and optical properties of the CQDs were examined using transmission electron microscopy (TEM), X-ray photoelectron spectroscopy (XPS), Fourier transform infrared spectroscopy (FTIR), heteronuclear single quantum coherence spectroscopy (2D-HSQC NMR), and photoluminescence (PL) measurements. Furthermore, the CQDs showed high sensitivity to Cu^2+^ ions, with detection limits of 0.059 µM for CQDs from tomato fruit and 0.048 µM for those from the stem.

## Materials and methods

### Chemicals and reagents

Tomato biomass (fruits and stems) is provided as a gift by the tomato producer Lušt d.o.o., located in Prekmurje, Slovenia. The biomass materials are properly prepared before use (washed, air-dried, and ground into a fine powder). The dopant, m-aminobenzoic acid, is purchased from Fluorochem, while hydrochloric acid (37% aqueous solution) is obtained from Pregl Chemicals, and NaOH (p.a.) is acquired from Sigma-Aldrich.

### Synthesis of Tomato CQDs

Tomato CQDs were prepared using a two-step, eco-friendly hydrothermal method. First, 0.3 g of biomass (fruit or stem) and 30 mL of de-ionized (DI) water were mixed and sonicated in a water bath for 30 min. After sonication, 0.3 g of *m*-aminobenzoic acid was added with constant stirring. The mixture was then heated at 90 °C for 1 h with continuous stirring. Afterwards, the heating was stopped, the reaction mixture was cooled to room temperature, and filtered through a 0.45 μm nylon filter. The filtrate was transferred into a Teflon-lined autoclave (50 mL) and heated at 200 °C for 12 h. Following the reaction, the solution was cooled to room temperature, sonicated for 10 min, and further filtered through a 0.22 μm filter. The resulting filtrate was dialyzed in a dialysis bag (500–1000 Da) against DI water for 2 days. The aqueous solution of dialyzed CQDs was stored as a liquid at 4 °C or freeze-dried at − 60 °C for 2 days and characterized as a powder. The samples are referred to as CQD F for CQDs made from tomato fruit and CQD S for those from dried tomato stems. The concentration of both samples after purification was 1 mg/mL.

### Characterization techniques of Tomato CQDs

The detailed characterization is given in the Supplementary file.

## Results and discussion

### Structure of CQD F and CQD S

**Fig. 1:**
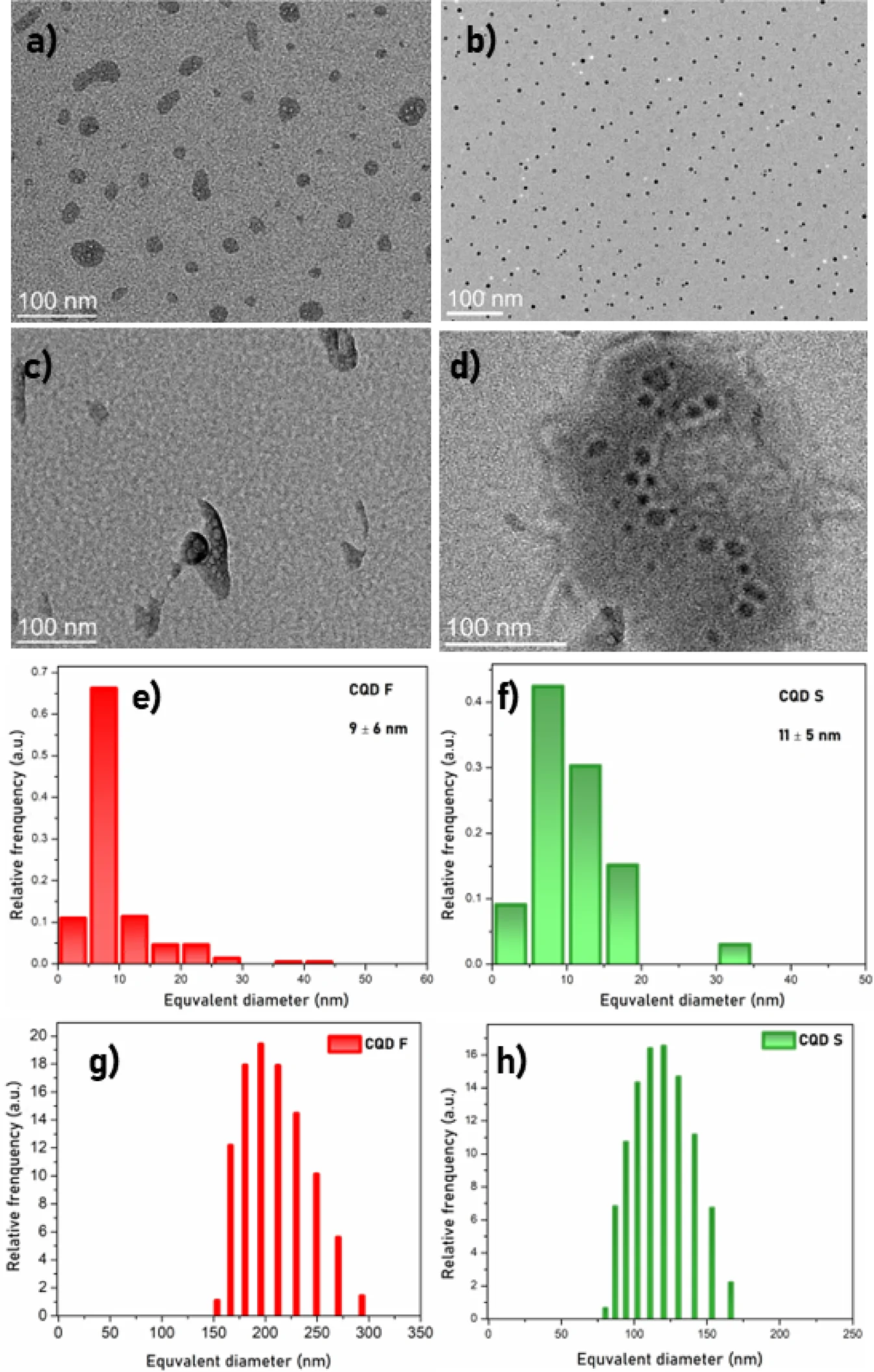
TEM images of: **a)**,** b)** CQD F, **c)**,** d)** CQD S, **e)** Equivalent diameter distribution of CQDs F (N total = 200, equivalent diameter with a standard deviation of the CQDs = 9 ± 3 nm), **f)** Equivalent diameter distribution of CQDs S (N total = 200, equivalent diameter with a standard deviation of the CQDs = 11 ± 5 nm), DLS analysis in water of **g)** CQD F and **h)** CQD S.

Both colloids, CQD F and CQD S, have nanoscale dimensions, with nanoparticles ranging from a few nanometers to 40 nm in diameter (Fig. 1a-d). In both colloids, the dominant nanoparticles have a diameter of around 10 nm (Figs. 1e and f). These particles form agglomerates in water, with average sizes of 208 ± 29 nm for CQD F and 118 ± 20 nm for CQD S (Fig. 1g, h). The deviation between TEM and DLS measurements is widely observed in carbon quantum dots dispersed in water, where TEM reflects the size of primary nanoparticles, while DLS measures the hydrodynamic diameter of solvated and weakly aggregated CQDs^35^. This aggregation originates from intermolecular interactions between surface functional groups (such as hydrogen bonding and electrostatic screening at mildly acidic pH), which promote the formation of reversible CQD clusters in aqueous dispersion^36,37^. After 6 months of storage, the samples remain transparent, with the same emission intensity and with slightly increased aggregation (Figure S1).

The native colloids are highly stable, with streaming potentials of -35.0 ± 0.4 mV at pH 4.65 (CQD F) and − 35.7 ± 0.6 mV at pH 5.3 (CQD S) (Figure S2). This aligns with previous studies, where a highly negative potential in a slightly acidic environment indicates a strongly anionic surface mainly caused by deprotonated oxygen groups like –COOH and –OH^38^.

**Fig. 2:**
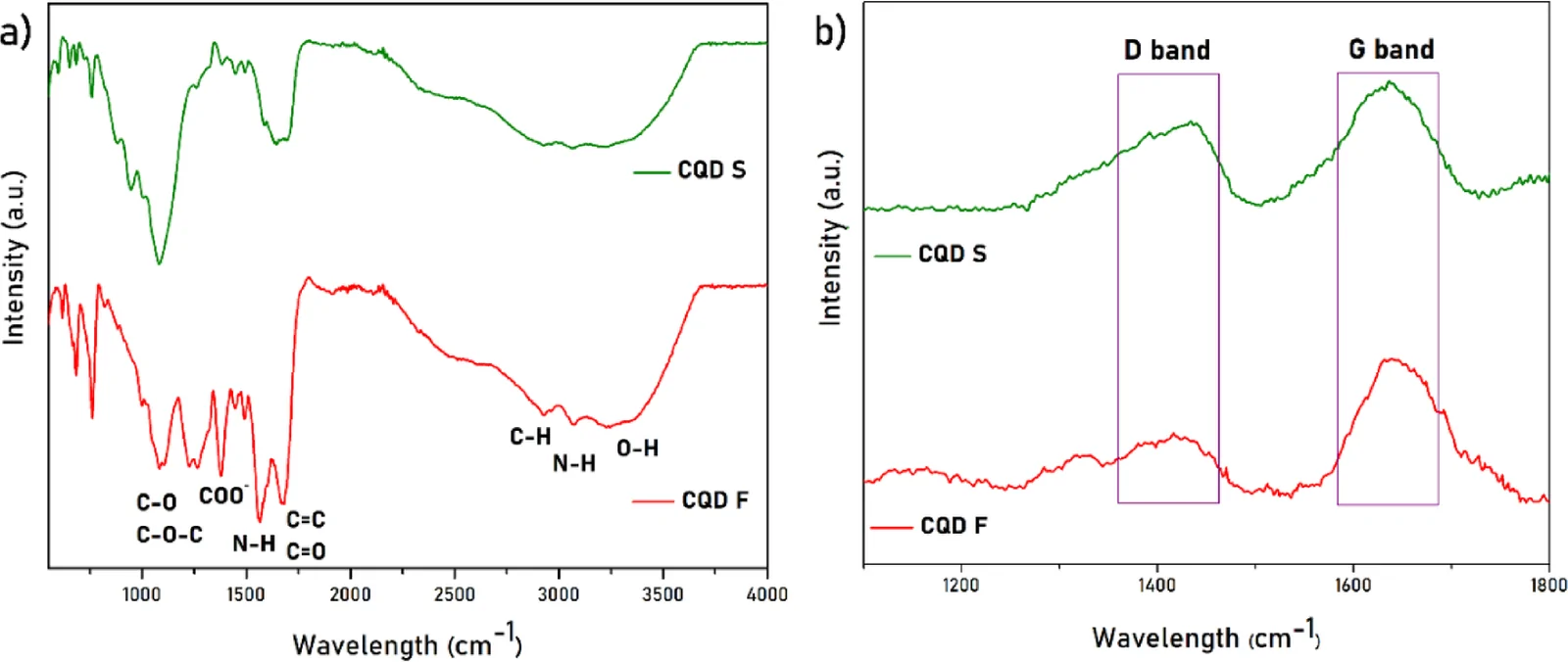
**(a)** FTIR spectra of CQD S and CQD F, and **(b)** Raman spectra of CQD S and CQD F samples.

FTIR spectra showed typical vibrations for N-doped biomass CQDs (Fig. 2a)^39,40^. The characteristic broad vibration around 3291 cm^− 1^ is attributed to OH functional groups, while the weak vibration at 3085 cm^− 1^ is related to the N-H group. A weaker peak around 2942 cm⁻¹ suggests the presence of C-H stretching. Additionally, the absorptions at 1668 cm^− 1^, and 1570 cm^− 1^ can be attributed to C = O, C = C, and N-H stretching. The vibration at 1383 cm^− 1^ is related to COO^−^ functional groups, while the sharp vibration at 1081 cm^− 1^ is associated with C-O and C-O-C vibrations. Raman analysis is presented in Fig. 2b. Two typical characteristic bands are observed. The G band (~ 1600 cm⁻¹) represents the E_2g_ in-plane vibration of sp² carbon (graphitic domains), while the D band (~ 1400 cm⁻¹) is a disorder/defect-activated mode (edges, vacancies, sp³ sites, heteroatoms). Slightly shifted D and G bands are in accordance with the literature^41^. Their ratio (I_D_/I_G_) quantifies the defect density and the graphitization of the samples.

For CQD S, the ratio ID/IG is 0.72, and for CQD F, it is 0.49, indicating that CQD S has a higher defect or functional group density and smaller sp² domains than CQD F. The ratios obtained are consistent with the literature and are typical for partially graphitized or moderately oxidized biomass carbons^42–44^.

**Fig. 3:**
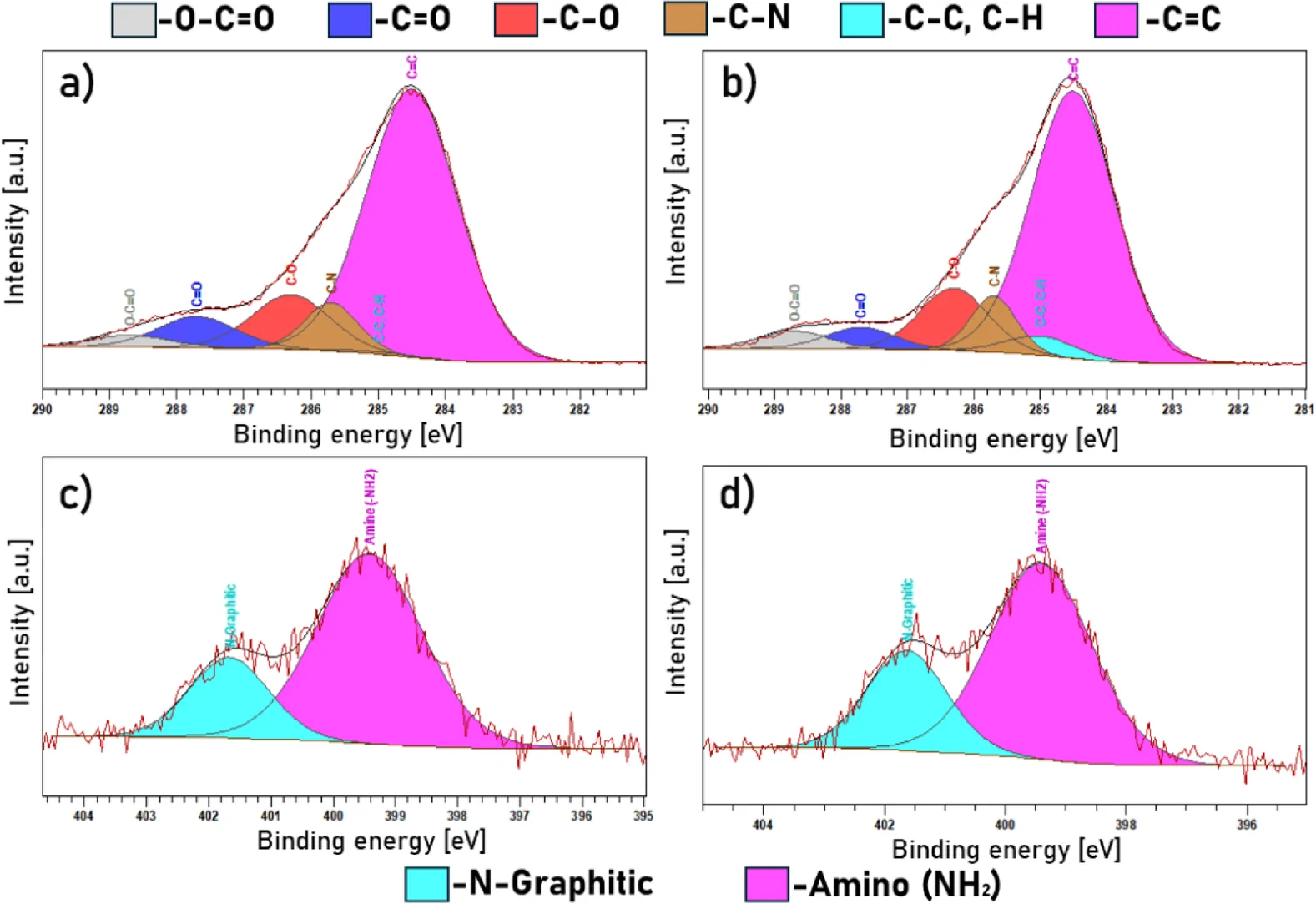
C1s and N1s Core levels XPS spectra of **a)**, **c)** CQD F, and **b)**, **d)** CQD S.

XPS analysis offers deeper insights into the surface functionalities of the prepared CQD F and CQD S samples. Semiquantitative elemental analysis of survey spectra (Figure S3) shows that both samples contain the same main surface elements (C, N, O), but their detailed surface compositions differ. Besides these elements, CQD S also contains Si, P, S, and Cl, whereas CQD F contains Si and only small amounts of P and S, with no detectable Cl. The detailed C1s and N1s core-level spectra are shown in Fig. 3. Both samples show a similarly high concentration of *sp*^*2*^–hybridized carbon due to carbonization (Supplementary file), but they differ significantly in their *sp*^*3*^ carbon content: CQD S contains about 4% *sp*^*3*^–hybridized carbon, whereas CQD F has almost none. CQD F also exhibits a higher proportion of C–O and C = O groups, indicating the presence of quinone-like functionalities on the surface of the carbon quantum dots. Both samples additionally have surface carboxyl (–COOH) groups. Furthermore, nitrogen is incorporated into the carbon lattice of both CQDs in similar proportions, with roughly 75% as amino nitrogen and about 25% as aromatic nitrogen.

### Mechanistic insights into the formation of CQD S and CQD F

To elucidate the reaction mechanism underlying the multi-step synthesis of CQDs, the reaction pathway and intermediates formed at each stage were systematically investigated. Particular emphasis was placed on NMR and FTIR spectroscopy to provide detailed insight into the chemical composition and structural evolution of the material.

**Fig. 4:**
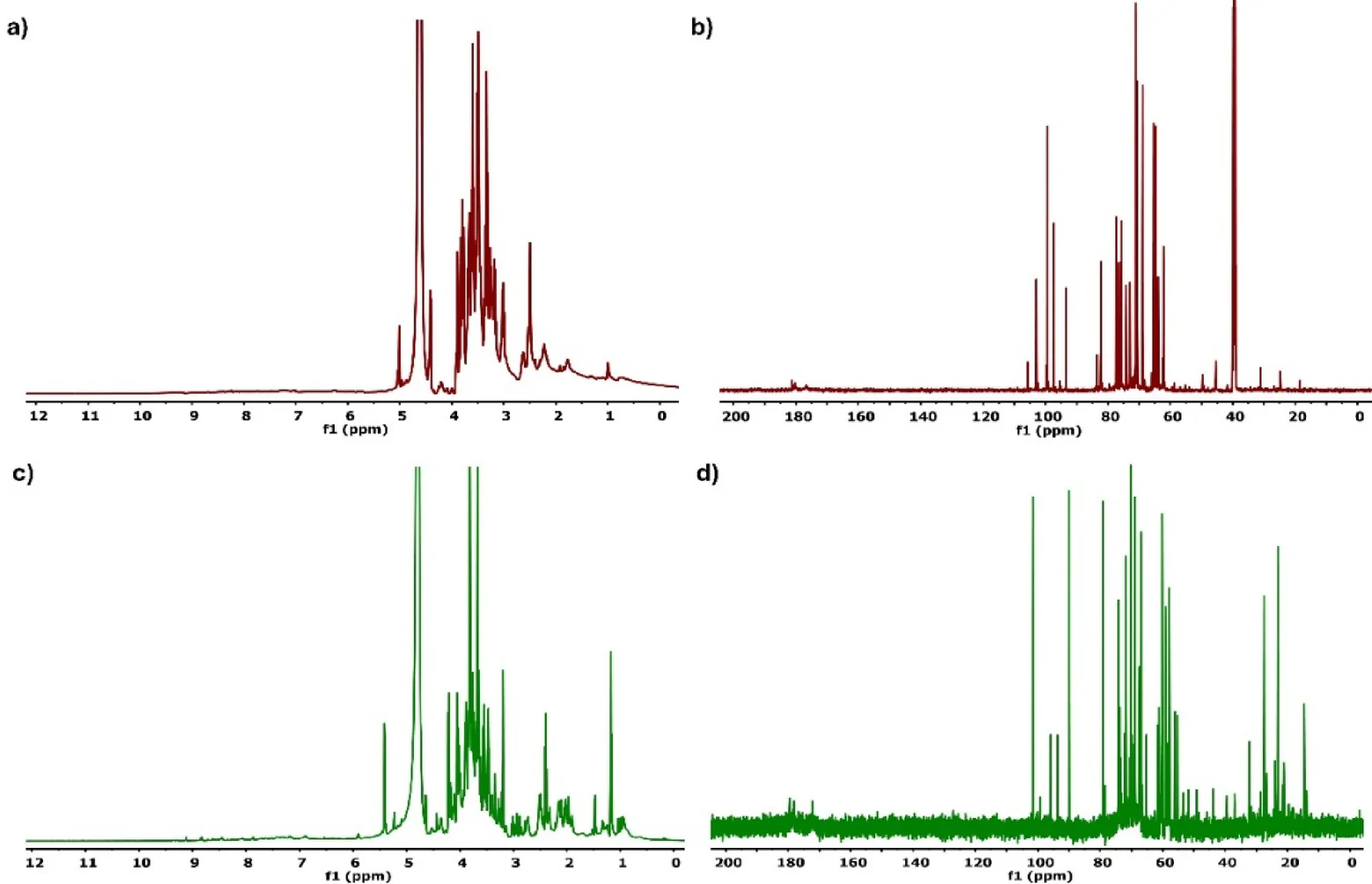
NMR spectra of tomato biomass extracts **(a)** ^1^H NMR spectrum of a tomato fruit biomass, **(b)** ^13^C NMR spectrum of a tomato fruit biomass, **(c)** ^1^H NMR spectrum of tomato stem biomass, **(d)** ^13^C NMR spectrum of a tomato stem biomass.

During the initial step, carried out in Milli-Q water with 30 min of ultrasonic treatment, the process is dominated by the extraction of low-molecular-weight compounds from the biomass. As the extracts were aqueous, the NMR spectra were recorded in deuterated water (D₂O). NMR analysis shows that the extracts obtained from both tomato fruit and stem biomass are predominantly composed of compounds with sp³-hybridized carbon atoms. Analysis of the integral ratio between the aliphatic and aromatic regions indicates that aromatic protons account for approximately 6% of the total steam biomass extractives and 4% of the aqueous extractives of fruit biomass (Fig. 4a and b). These results indicate that, under such mild conditions, mainly small molecules embedded within the biomass matrix – either associated with or located between the polymeric constituents such as lignin, cellulose, and hemicellulose – are selectively released. The absence of harsher conditions prevents significant degradation of these structural polymers, which is consistent with the observed selectivity of the extraction process^45^.

The ^13^C NMR analysis further confirms the predominance of aliphatic structures and reveals the presence of unsaturated moieties and carbonyl functionalities, highlighting the chemical diversity of the extracted compounds (Fig. 4c and d). The spectra further revealed the presence of carbonyl carbon atoms resonating in the range of 170–185 ppm in extracts obtained from both biomasses. Based on the chemical shifts observed in the ^13^C NMR spectra, it can be concluded that the structures predominantly contain hydroxyl, ether, and ester functional groups. To support the structural analysis, two-dimensional NMR spectroscopy was performed, revealing that carbon atoms resonating in the range of 60–108 ppm are correlated with protons resonating between 3.0 and 4.5 ppm (Figure S4). Comparison of all recorded NMR spectra further indicated that the majority of the carbon atoms within the structure are protonated.

Furthermore, FTIR analysis revealed diferencesbetween the spectra of the initial biomass and the corresponding extracts, particularly in the case of tomato fruit biomass (Figure S5). These differences can be attributed to the complex and rigid structure of the biomass, which consists of polymeric components such as cellulose, hemicellulose, and lignin, together with low-molecular-weight compounds present in the extractives. Since tomato fruit biomass contains a lower proportion of extractable compounds, the FTIR spectra of the raw biomass and the extracts differ more substantially. In contrast, tomato stem biomass contains a higher fraction of extractives, resulting in greater similarity between the spectra of the initial biomass and the obtained extracts. This observation is consistent with the extraction yields: approximately 60% for tomato fruit biomass and 22% for tomato stem biomass present extractives relative to the initial biomass mass (Table S1). The higher extraction yield obtained for fruit biomass indicates a greater abundance of readily extractable compounds compared to steam biomass. The results indicate that the extractable compounds are already highly accessible within the biomass matrix, making the application of ultrasound largely unnecessary for their release. Instead, ultrasound primarily facilitates mixing and homogenisation of the reaction mixture during the extraction process. In addition to the nearly identical extraction yields, if we compare ultrasound treatment of excraction and solely water extraction, without ultrasound, (Table S1), the NMR spectra were also highly comparable, indicating that the same compounds were present in the extracts irrespective of whether ultrasound treatment was applied (Figure S6). These results indicate that the first reaction step primarily produces low-molecular-weight compounds, including sugars, amino acids, organic acids, and simple aromatic species (in a very small proportion), which serve as key precursors for subsequent CQD formation. During the extraction of tomato fruit biomass, an interesting phenomenon was observed: the solid residue became highly sticky, suggesting a significant pectin content in the biomass^46^. Although tomato fruit biomass contains both soluble and insoluble pectic fractions, only one type was detected in the final extract^47^. In contrast, the intense red colour of the extract indicates the possible presence of various pigments. A possible candidate molecule is lycopene; however, it is insoluble in water^48^; other pigments might be present.

**Fig. 5:**
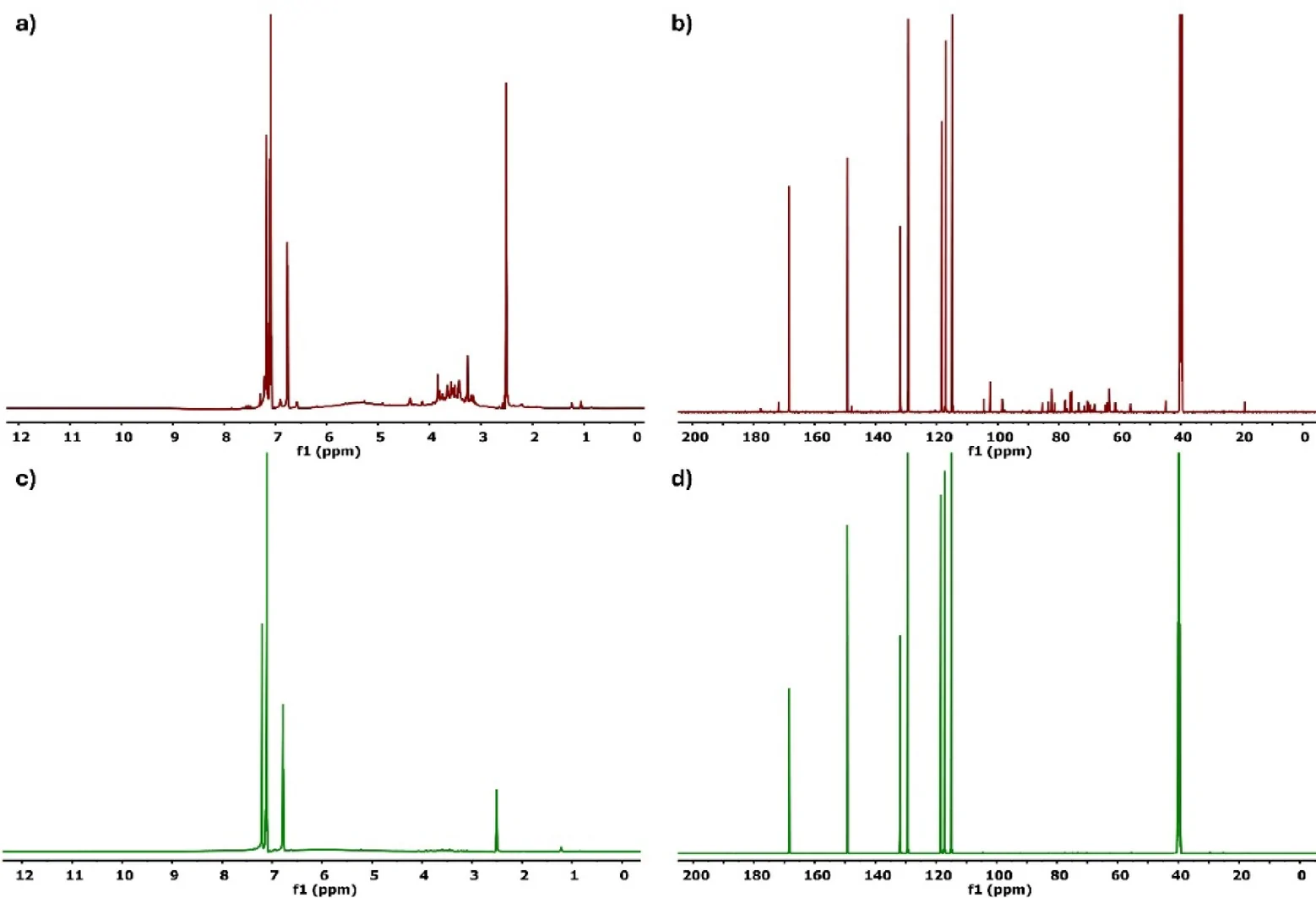
^1^H and ^13^C NMR spectra of tomato biomass extractives after reaction with 3-aminobenzoic acid **(a)** ^1^H NMR spectrum of a tomato fruit biomass extractives, **(b)** ^13^C NMR spectrum of a tomato fruit biomass extractives, **(c)** ^1^H NMR spectrum of tomato stem biomass extractives, **(d)** ^13^C NMR spectrum of a tomato stem biomass extractives.

NMR analysis of the second reaction stage confirms the presence of signals attributable to the 3-aminobenzoic acid dopant, as well as those from compounds formed in the initial step (Fig. 5). As the dopant 3-aminobenzoic acid exhibits limited solubility in aqueous media, the NMR spectra were recorded in DMSO-d6. Notably, no significant changes in chemical shifts are observed for either the dopant or the extract-derived signals compared to the spectra of pure 3-aminobenzoic acid (Fig. 5, Figure S7) and the first-stage products. This suggests that no chemical transformation occurs during this stage. The reduced intensity of signals associated with extract-derived species reflects their lower relative abundance compared to the added dopant. This effect is particularly evident in samples derived from tomato stem biomass, where mass of residue after extraction is lower then in fruit-derived residue (Table S1). The increase in product mass, and consequently in reaction yield, can primarily be attributed to the incorporation of the dopant. In both cases, the mass difference between the extract and the product obtained after the second reaction stage ranged from 234 to 247 mg. The slightly lower increase compared to the initial mass of the added dopant is likely due to losses during product isolation, as a portion of the dopant dissolved in the aqueous phase and was subsequently removed with the filtrate during filtration. To further verify the formation of more complex chemical products, a 2D HSQC spectrum was also recorded (Figure S8). The spectrum again indicated only the mixing of the biomass extract and the dopant, with no evidence of new product formation arising from chemical reactions.

Consistent with the NMR results, the FTIR spectra obtained after the second stage largely correspond to a superposition of the individual spectra of the dopant and the extracted compounds, further indicating the absence of new covalent bond formation or significant chemical transformations at this stage (Figure S5). This effect is even more pronounced for tomato fruit biomass. In contrast, for tomato stem biomass, the characteristic peaks of the dopant dominate the spectrum, effectively masking the signals from the extract. A similar trend was observed in the NMR spectroscopic analysis.

**Fig. 6:**
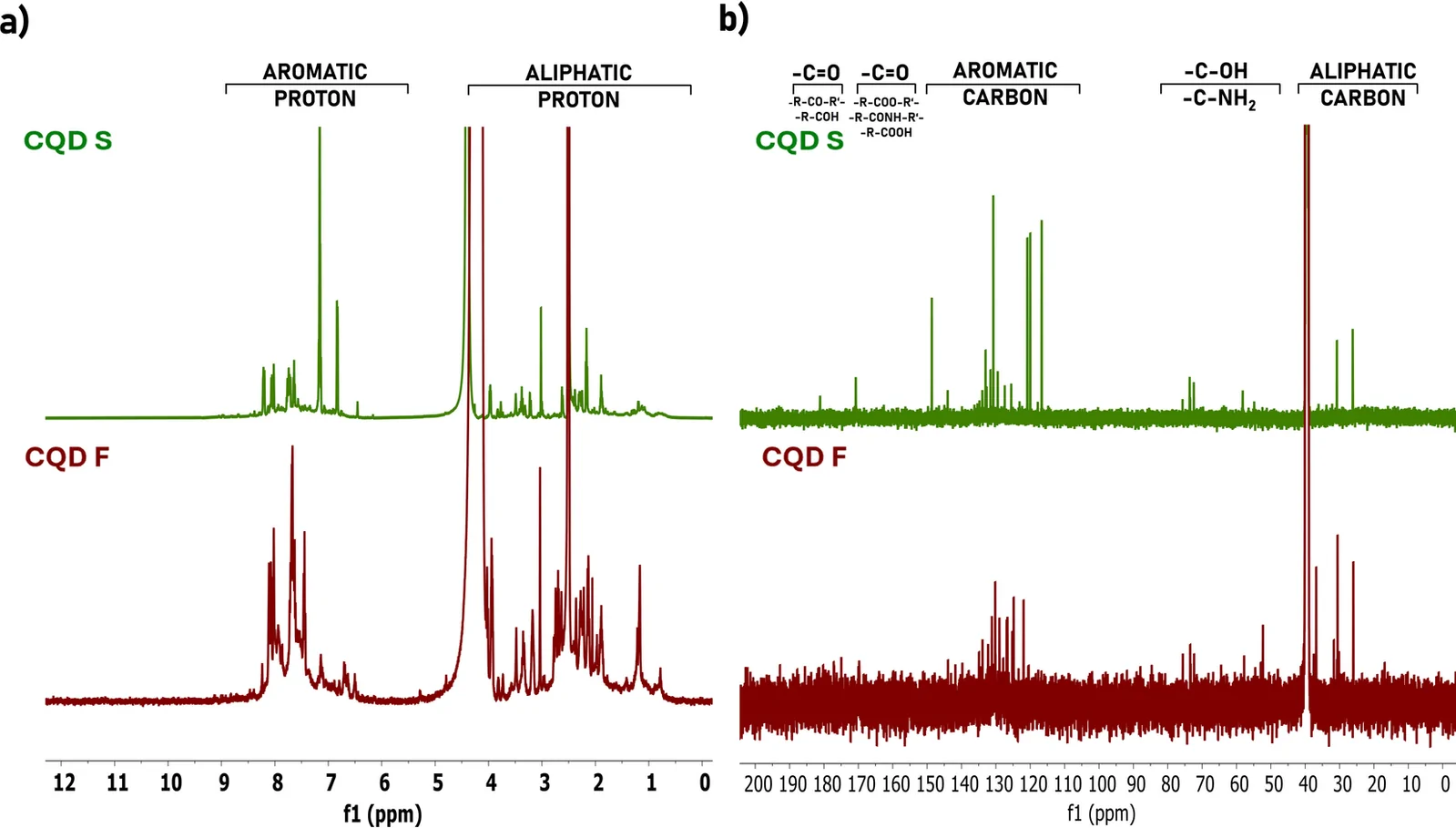
**(a)** ^1^H NMR, and **(b)** ^13^C NMR spectra of CQD S and CQD F.

Based on all the analyses performed, it can be concluded that no chemical reaction occurs under the conditions of the second-order process. Instead, at most, weak physical interactions are formed between the dopant and the molecules present in the extract. In contrast, substantial changes are observed when comparing the second-stage NMR spectra with those of the final products (CQD S and CQD F) (Fig. 6). A one-dimensional proton NMR analysis confirmed a highly heterogeneous material structure and the presence of both aromatic and aliphatic proton environments in the CQD samples (Fig. 6a). In both ^1^H NMR spectra of the synthesized CQDs, protons with relatively high chemical shifts (up to 8.5 ppm) are observed. These downfield shifts are typical of protons near electron-withdrawing groups, such as carbonyl or carboxyl groups, indicating a high level of oxidation and heteroatom incorporation. This aligns with expected biomass carbonization pathways, where oxidized aromatic structures form through hydrothermal carbonization. Conversely, in both cases, protons with relatively low shifts around 1 ppm are also present, suggesting that the structure includes alkyl groups without bound heteroatoms. Comparison of the spectra clearly indicates compositional differences between the two CQDs, most likely due to the distinct chemical profiles of the respective starting biomasses.

To gain further insight into the structural complexity of the synthesised CQDs, we analyzed the ¹³C NMR spectra (Fig. 6b). The ¹³C NMR spectra of both synthesised CQDs display signals corresponding to aliphatic and aromatic carbon environments, as well as resonances attributable to carbonyl carbons. However, no signals characteristic of ketone or free aldehyde functionalities are observed, which is consistent with the ¹H NMR spectrum, where there is also no peak for the aldehyde proton (Fig. 6b). Although the ^13^C NMR spectra were acquired using a high number of scans (15,000), a relatively high level of noise is still evident compared to the other NMR techniques discussed. As similar noise was observed even with increased sample amounts, it is most likely intrinsic to the nature of the samples. These systems appear to possess a rigid and heterogeneous carbon framework, which is typically associated with short carbon relaxation times, ultimately leading to reduced signal quality. Due to the nature of the sample, the resulting spectra may exhibit slight variations depending on the measurement conditions employed. These structural changes are also reflected in the differing solubility of the products obtained at each reaction stage. Because D₂O was used as the solvent in the first reaction step and DMSO in the second step, DMSO-d₆ supplemented with D₂O was employed as the NMR solvent for the final reaction step. As part of the NMR analysis, the same sample was measured repeatedly under identical experimental conditions at different time intervals. No significant spectral differences were observed, confirming the stability of the samples over time. During the analysis, it was also found that freeze-drying of the CQD samples prior to NMR measurements is preferable to solvent removal by rotary evaporation, as the latter may induce structural changes in the CQD samples.

The appearance of new signals, including in the aromatic region, provides clear evidence of chemical transformations occurring during the hydrothermal treatment. This is further supported by FTIR analysis, where the spectra of the final materials no longer reflect a simple superposition of the initial components but instead indicate the formation of new functional groups (Fig. 2 and Figure S5).

.

**Fig. 7:**
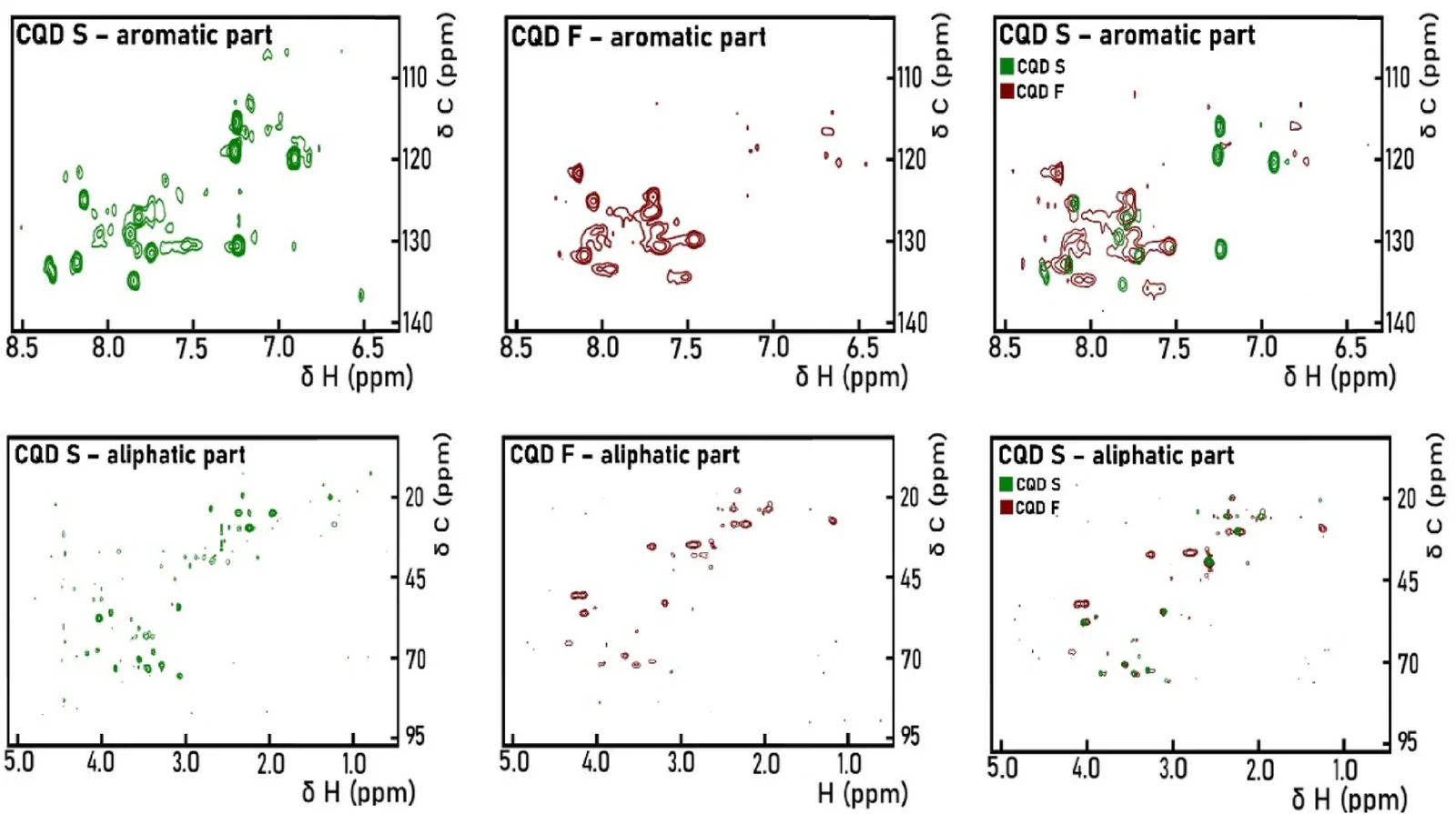
2D-HSQC NMR spectra of CQD F and CQD S.

Since the ^13^C NMR spectrum displays all carbon atoms in the structure of the synthesised CQDs and is therefore quite complex, we focused only on those carbon atoms that also have a proton attached. We achieved this by analysing 2D HSQC NMR (which, in this case, is a more sensitive technique than ^13^C NMR spectroscopy), which further confirmed contributions from both aliphatic [¹H: 1.1–4.2 ppm / ¹³C: 16.0–76.2 ppm] and aromatic [¹H: 6.6–8.4 ppm / ¹³C: 113.8–135.9 ppm] moieties, verifying the heterogeneous molecular architecture of the carbon dots (Fig. 7). Although the two CQDs originate from chemically distinct biomass, both materials display comparable aromatic and aliphatic structural domains. As the samples underwent dialysis to remove low-molecular-weight molecules, these observations suggest that aromatic units are covalently interconnected with aliphatic chains in both systems, forming integrated hybrid nanostructures.

The 2D HSQC NMR reveals the presence of well-defined aromatic nuclei in the structure of CQDs. This spectral consistency suggests specific structural motifs, likely resulting from partial depolymerization of macromolecular precursors during carbonization, which produces smaller and more uniform aromatic fragments. Alternatively, these distinct spectral features may stem from the preferential transformation of simpler aromatic compounds naturally found in the biomass feedstock. Similar orderliness and defined cores are also seen in the aliphatic region of the 2D HSQC NMR spectrum. In both spectra, certain signals, in both the aliphatic and aromatic regions of the NMR spectrum, overlap, indicating similar structures in CQDs. This implies that these signals could be linked to the aromatic cores of the dopant, despite the vastly different compositions of tomato stem and tomato fruit biomass. Conversely, signals at higher chemical shifts show different patterns.

To determine the origin of molecules from individual biomass, we compared the ratios of integrals between the aliphatic and aromatic regions for both synthesized CQDs, excluding the DMSO-d6 solvent and the aromatic nuclei range attributable to the dopant. The results showed that this ratio is lower for CQD F, which is unexpected, as tomato stem biomass contains approximately 13.5% lignin, a key source of aromatic units^49^.

Comparison of the NMR spectra of the final products with that of the dopant (Figure S9), acquired under identical experimental conditions using DMSO-d₆ supplemented with D₂O as the solvent, reveals clear differences in the CQD formation process. For CQD S, more intense signals attributable to the dopant are observed (Figure S10), whereas these signals are considerably less pronounced in CQD F. As prolonged dialysis was performed for both samples and the efficiency of purification was monitored through conductivity measurements of the aqueous dialysis medium, it can be inferred that the dopant plays a more prominent role in CQD S and is most likely incorporated through physical interactions, as no significant chemical shift changes are observed in the NMR spectra. In contrast, for CQD F, the extractive compounds themselves appear to provide sufficient precursors for CQD formation, or alternatively, the dopant may be incorporated through chemical bonding. We as well prepared CQD from only the dopant 3-aminobenzoic acid, with the same conditions of synthesis and purification as prepared CQD S and CQD F. At the end of purification, we obtained slightly luminescent colloid (Figure S11), with yield of only 0.7 mg of 3-ABA CQDs. So, the dopant 3-aminobenzoic acid is preferently condensated and carbonised with steam and fruit extractives, rather then a formation of carbon nanoparticles constituited from only its molecules.

The results therefore suggest that lignin is not the primary contributor to aromatic domains in either CQD. The reaction conditions used in the first stage of synthesis are much milder than those typically used for lignin extraction. Classical Organosolv delignification requires high temperatures, strong acid catalysts, and often organic co-solvents. To estimate the lignin yield, we performed Organosolv delignification under similar aqueous conditions (120 °C and dilute HCl). Under these conditions, we isolated about 1% lignin relative to the biomass weight. Since we used a weaker acid, the lignin yield was even lower. Additionally, Organosolv processing produces water-insoluble lignin, which would be removed during filtration in our method. This strongly supports the conclusion that lignin fragments do not remain in the final CQDs. Instead, the formation of the nanoscale carbon cores appears to be mainly driven by aromatic and aliphatic molecules in the water-soluble extractives. These account for approximately 22% of tomato stem biomass and 40–50% of tomato fruit biomass, aligning with the observed differences between the two CQD samples^2^.

Considering the above, we can conclude that, in the case of CQD S, condensed aromatic domains are most likely formed from native phenolic compounds such as caffeic acid, chlorogenic acid, gallic acid, and rutin^2^. On the other hand, CQD F comes from biomass that is richer in lipids, proteins, and pectin, while still containing phenolic metabolites. This variation in composition causes multiple overlapping signals in both the aromatic and aliphatic spectral regions^40^. These components can also affect emissive behavior. Additionally, pectic polysaccharides, which are rich in hydroxyl and carboxyl groups, are found only in tomato fruit and can undergo thermal transformation into aromatic species when subjected to hydrothermal treatment at 200 °C^43^.

**Fig. 8:**
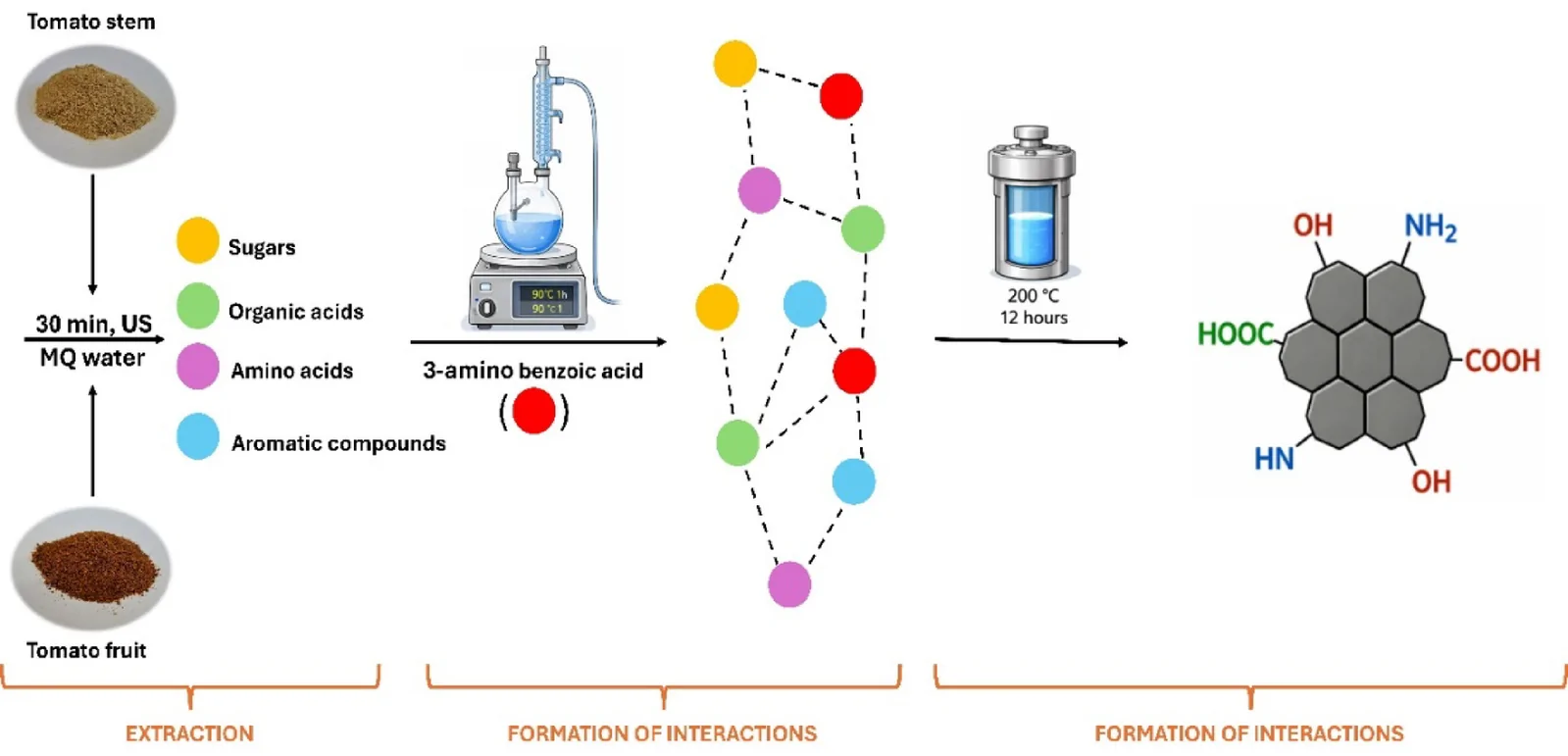
Schematic illustration of the proposed reaction mechanism for the synthesis of CQDs from tomato fruit and stem biomass.

Based on these observations across the individual stages, a reaction mechanism is proposed, as illustrated in Fig. 8.

The schematic illustrates a three-step process for the conversion of biomass into a functionalized carbon material. In the first step, ultrasonic treatment in Milli-Q water facilitates the extraction of water-soluble components, including sugars, organic acids, amino acids, and low-molecular-weight aromatic compounds. In the second stage, the introduction of 3-aminobenzoic acid leads to partial reorganization of the system through non-covalent interactions, such as hydrogen bonding and π–π stacking, without inducing covalent modification. The final stage involves hydrothermal treatment (200 °C, 12 h), during which condensation and aromatization processes occur, resulting in the formation of a stable carbonaceous network. This structure incorporates functional groups (–OH, –COOH, –NH₂) derived from both the extracted biomass components and the added dopant. If we take into account the total masses of the initial biomass and dopants, then the yield is 3% for CQD F and 4.3% for CQD S, respectively (Table S1).

### Optical properties of CQD F and CQD S samples

**Fig. 9:**
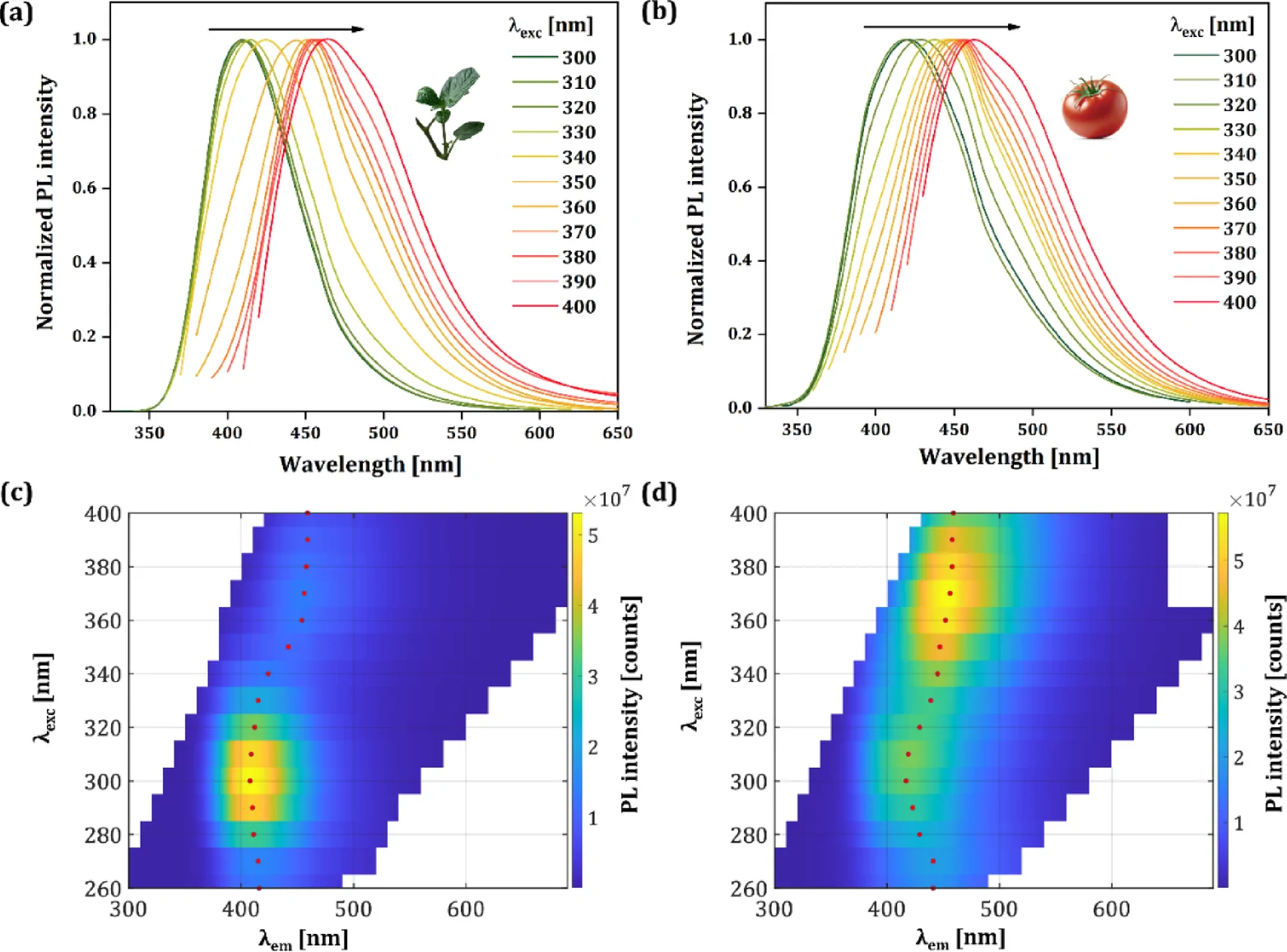
**(a)** normalized PL intensity of CQD S (1 mg/mL) at various excitations from 300 to 400 nm, **(b)** normalized PL intensity of CQD F (1 mg/mL) at various excitations from 300 to 400 nm, **(c)** 2D PL map of CQD S, **(d)** 2D PL map of CQD F.

The optical properties of the CQD F and CQD S were analyzed using photoluminescence (PL) spectra with excitation wavelengths ranging from 300 nm to 400 nm (Fig. 9). The normalized PL spectra of both samples show excitation-dependent emission behavior, characterized by a noticeable redshift in emission wavelengths as the excitation wavelength increases. Specifically, the emission peaks of CQD S and CQD F samples shift from 410 nm to 470 nm as the excitation wavelength varies from 300 nm to 400 nm (Fig. 9a and b, respectively). This behavior can be attributed to the inherent heterogeneity of the CQDs, which arises from differences in particle size, surface functional groups, and the presence of multiple emissive states. Smaller sp² domains within the CQDs tend to have larger band gaps, emitting at shorter wavelengths, while larger domains or surface-related trap states have lower energy gaps, resulting in red-shifted emission under longer-wavelength excitation^50^.

As previously discussed, doping with m-aminobenzoic acid introduces extra amino and carboxyl groups, creating new surface states that further expand the range of emissive centers. Additionally, the aggregation of CQDs in aqueous media (Fig. 1) can facilitate energy transfer between particles, promoting emission from lower-energy states and causing a red shift. This excitation-dependent behavior is typical of biomass-derived CQDs and reflects the complex interaction among core states, surface or defect states, and molecular fluorophores linked to the carbon network^22,40,51,52^. This tunable photoluminescence highlights the potential of these CQDs for selective sensing applications, where the excitation wavelength can be optimized to target specific analytes.

Figure 9c and d display a 2D PL map of CQD S and CQD F samples, respectively, showing clear excitation-dependent emission behavior. In this plot, the Y-axis indicates the excitation wavelength, the X-axis shows the emission wavelength, and the photoluminescence intensity is represented by a color scale, with blue indicating the lowest intensity and yellow representing the highest. These maps reveal that the highest emission intensity occurs at 300 nm excitation for CQD S and at 370 nm for CQD F. These excitation wavelengths were chosen for further fluorescence quenching experiments using Cu^2+^ ions.

**Fig. 10:**
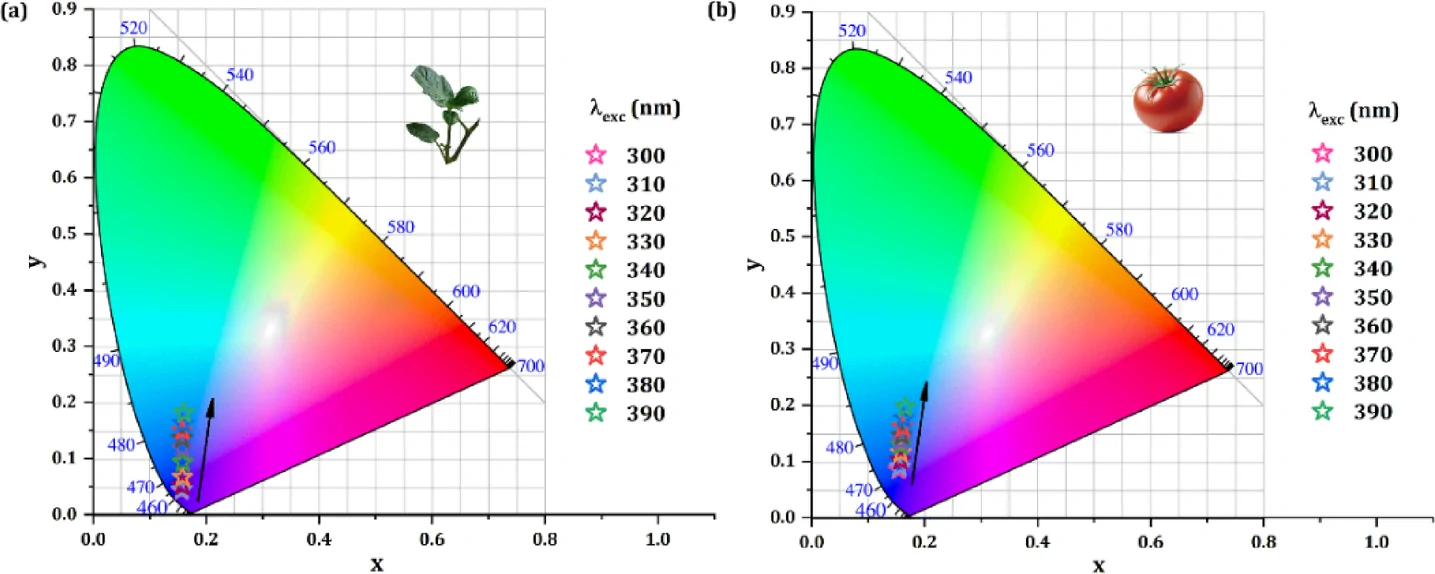
CIE 1931 chromaticity diagram of: **(a)** CQD S, and **(b)** CQD F.

The color properties of photoluminescent materials can often be described using the CIE 1931 chromaticity diagram, which shows perceived colors through chromaticity coordinates (x, y). To assess the photoluminescent behavior of the CQD S and CQD F colloids, their emission spectra were analyzed, and the corresponding chromaticity coordinates were plotted on the CIE diagram. Regardless of the excitation wavelength, the CIE coordinates for both CQD S (Fig. 10a) and CQD F (Fig. 10b) lie in the blue region of the diagram; however, as the excitation wavelength increases from 300 to 400 nm, the coordinates shift toward the green region. These excitation-dependent emission changes are typical for CQDs, arising from multiple emitting sites and surface states, further confirming their tunable photophysical properties.

The quantum yield (QY) of luminescent materials is a crucial parameter for potential applications because it indicates the optical efficiency of these materials. Different biomasses (fruit peels, juices, cellulose, proteins, etc.) have widely varying amounts of carbon, oxygen, and native heteroatoms; this variation affects carbonization pathways and surface groups, which significantly influence photoluminescence and QY. A comparison of QY for CQD S and CQD F with data from the literature is provided in Table 1. All selected materials were prepared hydrothermally with an N-dopant, where the incorporation of the N-dopant enhances the luminescent properties of the material.

**Table 1:** QY comparison for CQD S and CQD F with selected biomasses from the literature.

Biomass source	*N*-dopant	QY (%)	Reference
Lemon extract	Hydroxylamine	5.00	^53^
Tomato extract	Hydroxylamine	3.38	^53^
Oyster shell	Etilendiamine	1.50	^54^
Sheep manure	Glycine	10.63	^55^
Tomato steams	*m*-aminobenzoic acid	20.20	This work
Tomato fruits	*m*-aminobenzoic acid	3.80	This work

The obtained QY values show that tomato-derived CQDs, especially CQD S, are promising for various applications. The QY of 20.20% indicates that these biomass-based CQDs have moderate brightness, making them suitable for most sensing, imaging, or fluorescent labeling uses. However, they might not be enough for high-efficiency LEDs or applications needing extremely low-light detection.

#### Emission intensity quenching of CQD F and CQD S in the presence of Cu^2+^

**Fig. 11:**
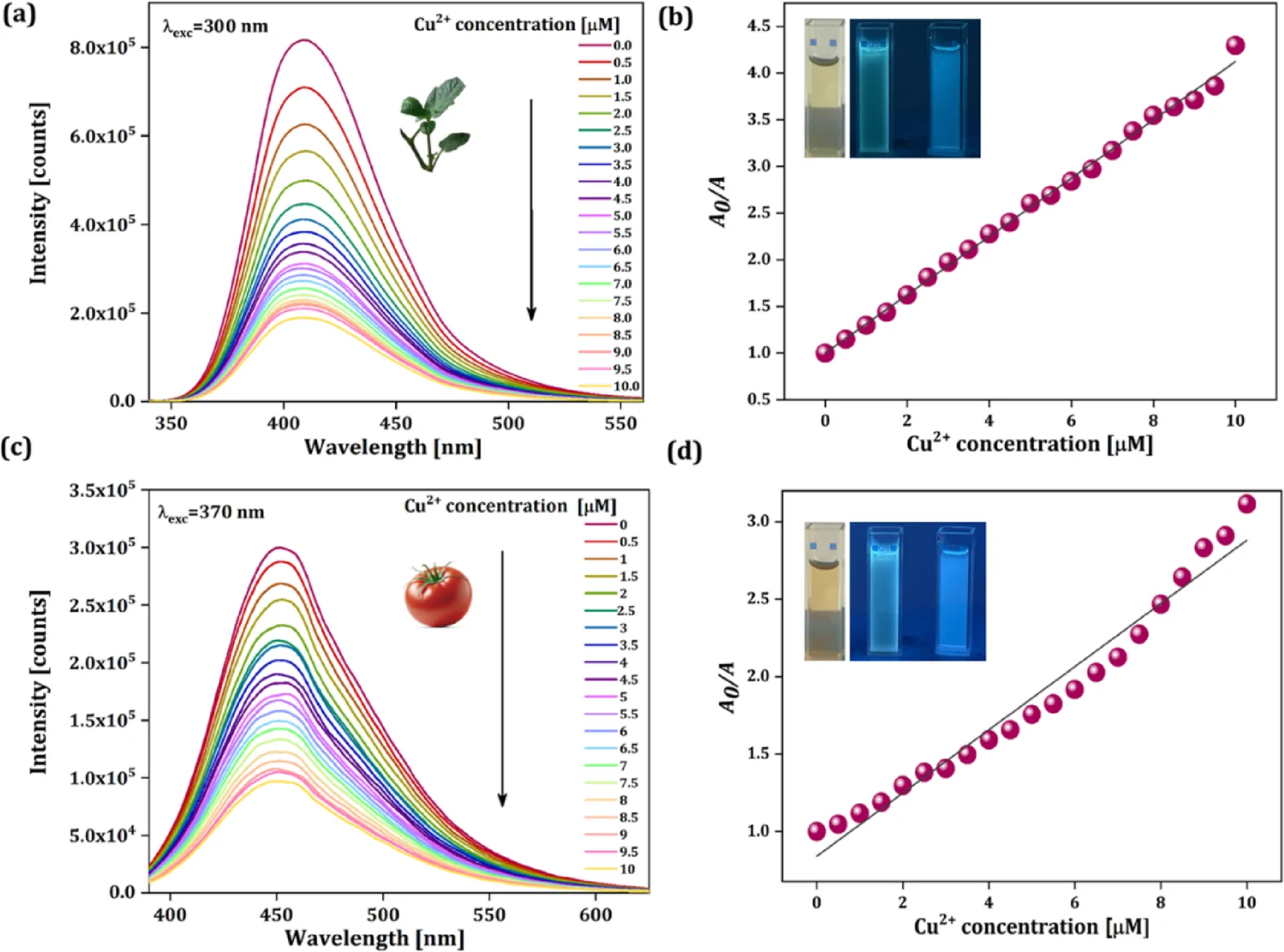
**(a)** CQD S PL emission intensity quenching in the presence of Cu^2+^ ions, **(b)** Stern–Volmer plot of the Cu^2+^-induced emission quenching of CQD S NPs, **(c)** CQD F PL emission intensity quenching in the presence of Cu^2+^ ions, **(d)** Stern–Volmer plot of the Cu^2+^-induced emission quenching of CQD F NPs. Visual representations of the quenching process are given as **insets** in Fig. 8b and d.

Figure 11a illustrates the quenching of emission from the CQD S sample when various concentrations of Cu^2+^ ions (0–10 µM) are added, recorded across the 340–560 nm range. A strong, broad emission peak around 410 nm is reduced by approximately 62% at a Cu^2+^ concentration of 5 µM, while the highest concentration used (10 µM) leads to a 77% reduction in emission intensity. Figure 11b shows a linear fit of the experimental data (integrated emission intensity versus quencher concentration) to the modified Stern–Volmer equation:

1 $$\:{A}_{0}/A=1+{K}_{SV}\left[Q\right]\:$$

where *A*_*0*_ and *A* represent the integrated emissions of CQD S sample in the absence and presence of Cu^2+^ ions, respectively; *Ksv* is the Stern–Volmer constant; and [Q] is the quencher concentration. A good linear relationship (the goodness-of-fit parameter R^2^ = 0.99, Table 2) is observed, with no deviation from the linear fit, which indicates that only dynamic quenching occurred in this concentration range of Cu^2+^ and that all CQD S particles were equally accessible to the quencher. Both samples were well dispersed, exhibiting a yellowish transparent solution under visible light and strong blue/green emission under UV irradiation (the quenching of CQD S and CQD F emission is shown as insets in Fig. 11b and d, respectively).

Figure 11c shows the quenching of the emission of CQD F sample upon adding different concentrations of Cu^2+^ ions (0–10 µM), recorded over the range 390–630 nm. One strong, broad emission peak around 460 nm is quenched by about 43% at a Cu^2+^ concentration of 5 µM, while the highest Cu^2+^ concentration used in this experiment (10 µM) decreased the emission intensity by 68%. Figure 11d displays a linear fit of the integrated emission intensity versus quencher concentration according to Eq. (1). A similar linear relationship (R^2^ = 0.97, Table 2) is seen for CQD F, with no deviation from the linear fit.

**Table 2:** Parameters of the Stern–Volmer fit (emission intensity vs. Cu^2+^ concentration) for CQD S and CQD F samples.

Sample	Linear range (µM)	σ	K_sv_ (µM^− 1^)	*R* ^2^
CQD S	1–10	0.005	0.31232	0.99
CQD F	1–10	0.004	0.20404	0.97

The limit of detection (LOD) was calculated using the following formula:

2 $$\:LOD=3\sigma\:/{K}_{SV}$$

where ϭ represents the standard deviation of the blank measurements, calculated from 50 recorded spectra. LOD values of 0.048 µM and 0.059 µM were obtained for CQD S and CQD F samples, respectively, indicating a slight difference in the sensitivity of these systems towards Cu^2+^, as shown in Table 3. In addition to our findings, Table 3 includes previously reported LOD values for Cu^2+^ ions with different QDs. It can be seen that LOD values for CQD F and CQD S samples are lower than or comparable to those previously reported. However, additional experiments are needed to fully assess the potential application of the obtained nanoparticles in Cu^2+^ detection.

**Table 3:** Comparison of QD probes for the detection of Cu^2+^ ions.

QD probe	LOD (µM)	Linear range (µM)	Reference
CDs from taurine	0.620	0.3–25.0	^30^
CDs	1.380	0.0–9.0	^31^
CdTe QDs	0.055	0.1–5.0	^56^
Si NP–CD nanoprobe	0.010	0.0–70.0	^32^
Cyst-CdS QDs	0.340	0.5–2.3	^57^
FNS-CDs	0.083	1.0–25.0	^33^
P-CQDs	0.032	0.0–260.0	^34^
Cu-*β*-Cd/N-CQDs	0.094	1–125	^58^
CQD F	0.059	0–10	This work
CQD S	0.048	0–10	This work

*Abbreviations*: CDs, carbon dots; CdTe QDs, cadmium telluride quantum dots; Si NP–CD, silicon nanoparticles with carbon dots; Cyst-CdS QDs, Cysteamine-capped cadmium sulfide quantum dots; FNS-CDS, fluorine-, nitrogen-, sulfur-doped carbon dots; P-CQDs, P-doped carbon quantum dots; Cu-β-Cd/N-CQDs, copper ion coordinated *β*-cyclodextrin nitrogen-doped carbon quantum dots.

**Scheme 1:**
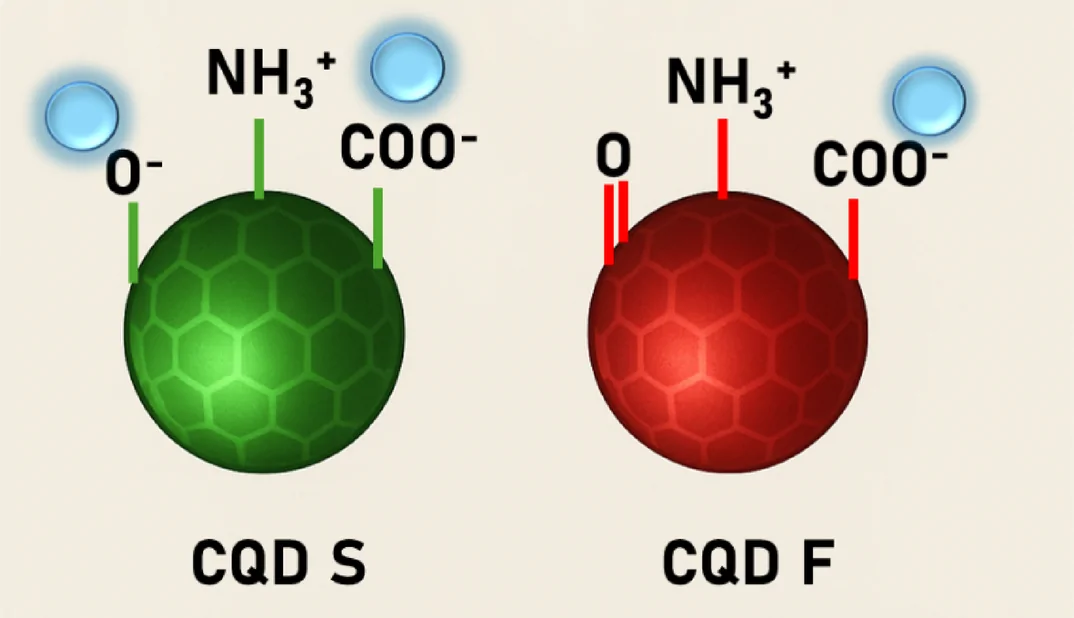
Representation of the distribution of functional groups on the surface of CQD S and CQD F with possible interaction with Cu²⁺ ions (presented as blue balls).

The fluorescence quenching of tomato-derived CQDs by Cu²⁺ is mainly controlled by static complex formation between surface functional groups (–COOH, –NH₂, –OH) and Cu²⁺ ions, with contributions from photoinduced electron transfer (PET) and possibly aggregation effects in aqueous media. CQD S showed an almost linear Stern–Volmer relationship, indicating a single dominant quenching pathway consistent with static complexation. This matches the XPS analysis, as the CQD S sample has a slightly higher concentration of functional groups (especially -OH and -COOH), which can react with Cu²⁺ (see Scheme 1). In contrast, fruit-derived CQDs showed weaker, more linear quenching. This behavior suggests the presence of multiple emissive sites with different accessibilities to Cu²⁺, likely caused by heterogeneous surface functionalization, particle size variation, or local aggregation. Interestingly, this sample has more carbonyl groups, which form only weak interactions with Cu²⁺. As a result, CQD S provides a predictable, linear sensing response (LOD = 0.048 µM), while CQD F offers slightly higher sensitivity (LOD = 0.059 µM) but displays quenching dependent on multiple sites dynamics. These findings demonstrate that the biomass source and surface chemistry critically determine both the quenching mechanism and analytical performance, offering a framework for designing CQD-based sensors with tailored sensitivity towards Cu²⁺.

#### Temporal response of emission of CQD S and CQD F samples in the presence of Cu^2+^ quencher

**Fig. 12:**
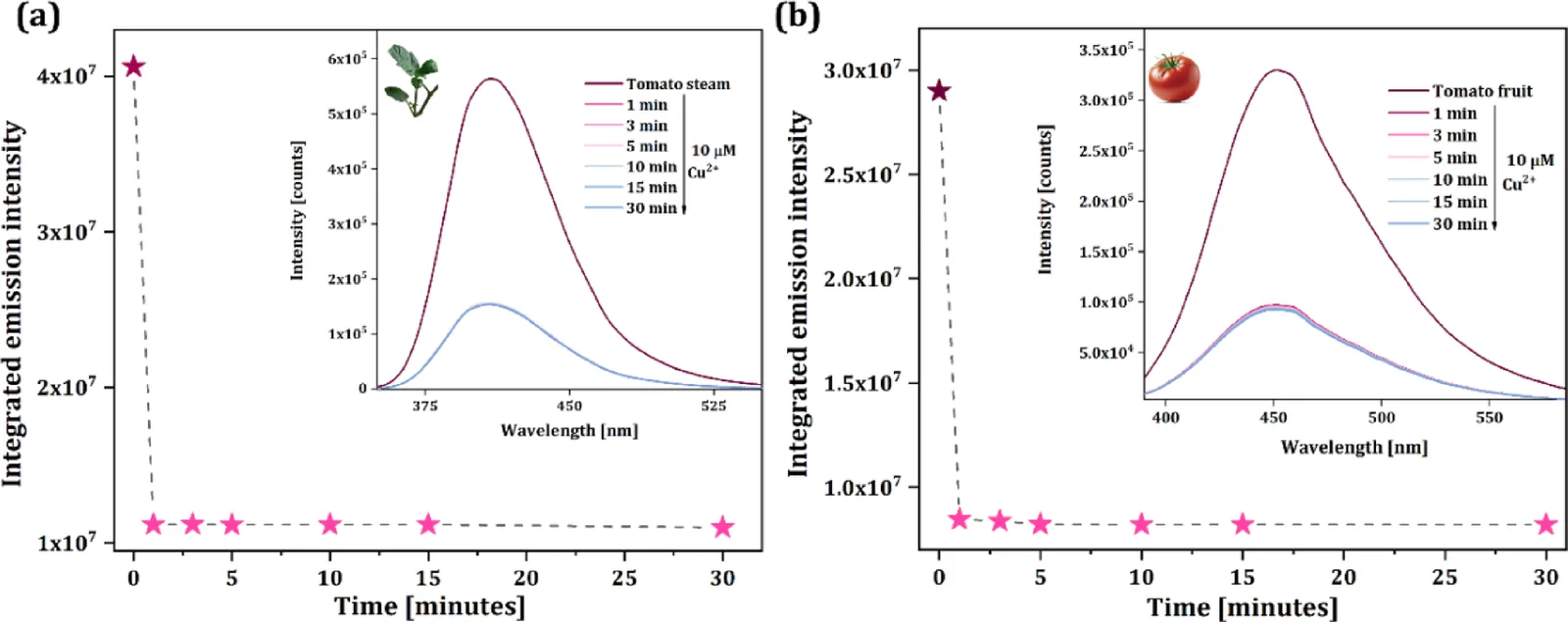
Temporal evolution of the emission intensity of **(a)** CQD S and **(b)** CQD F particles upon the addition of 10 µM of Cu^2+^ ions.

The inset of Fig. 12a and b shows the time-dependent emission spectra of CQD S and F recorded from 1 to 30 min after the addition of 10 µM Cu²⁺ ions. A rapid and significant drop in emission intensity was observed immediately after Cu²⁺ addition. Around 5 min later, the emission intensity stabilized, indicating that this time marked the achievement of equilibrium conditions for the formation of a stable, uniform dispersion of colloidal CQD F and S particles with copper ions. Each spectrum was measured 5 min after the addition of different copper ion concentrations.

#### Influence of ionic strength on fluorescence intensity of CQD S and CQD F samples

**Fig. 13:**
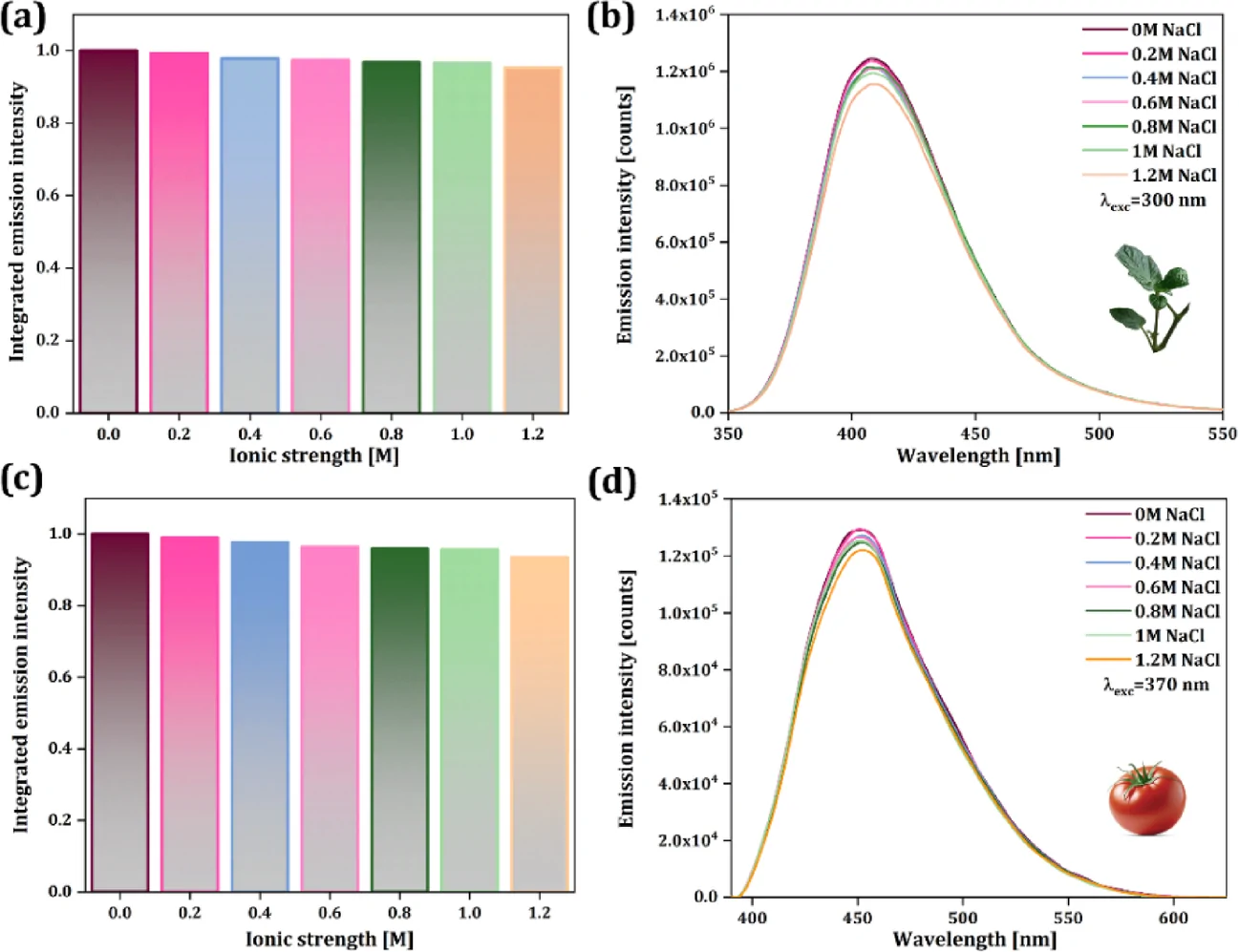
Integrated emission intensity in the presence of different ionic strengths of NaCl: **(a)** CQD S and **c)** CQD F, and CQDs PL response to various ionic strength values of NaCl, **(b)** CQD S and **d)** CQD F.

The influence of ionic strength on the fluorescence intensity of CQD S and CQD F was studied by changing the NaCl concentration from 0 to 1.2 M and measuring the fluorescence at fixed excitation wavelengths of 300 and 370 nm, respectively. As shown in Fig. 13, there is no noticeable decrease in PL intensity as ionic strength increases. This small change in overall emission can be attributed to the compression of the electric double layer around the CQDs, which reduces surface charge repulsion and may cause partial aggregation or change surface electronic states. Despite this minor effect, the CQDs from tomato maintained strong fluorescence even at high ionic strengths, demonstrating good stability and potential for use in complex or highly ionic environments [15].

## Conclusion

In our study, we prepared two types of colloidally stable carbon quantum dots from tomato biomass stems-CQD S and fruit-CQD F, respectively. Both materials are nanosized and mostly form aggregates in water, with highly negative surface charges. Raman analysis revealed that colloidal CQD S has a higher defect and functional group density and smaller *sp²* domains compared to CQD F. FTIR and XPS analyses confirmed the presence of expected functional groups on the surfaces of CQD S and CQD F, including hydroxyl, carbonyl, carboxyl, and two types of N-groups—amino and graphitic. Both colloids exhibit a similar high concentration of *sp*^*2*^-hybridized carbon due to carbonization; however, they differ in *sp*^*3*^ carbon content: CQD S contains about 4% *sp*^*3*^-hybridized carbon, whereas CQD F shows virtually none. NMR analyses demonstrated that both CQDs have heterogeneous structures made up of interconnected aromatic and aliphatic domains. Despite originating from chemically different biomasses, the structural properties suggest that water-soluble extractives, rather than lignin, are the main contributors to the aromatic cores. Overall, the results indicate that variations in biomass composition—particularly phenolic metabolites, lipids, proteins, and pectic polysaccharides—likely significantly influence the molecular structure and heterogeneity of the resulting CQDs. This study is also an initial investigation into the use of colloids derived from tomato biomass for potential Cu²⁺ detection. While the current work illustrates fundamental quenching behavior, further system optimization is needed. Selectivity studies and calibration curves with real samples have not yet been performed, but will be addressed in future research to thoroughly evaluate the system’s practical applications and analytical performance. We hope these future studies will provide deeper insights and help establish a robust platform for Cu²⁺ sensing.

## Supplementary Information

Below is the link to the electronic supplementary material.

## Data Availability

The data bill be available upon request.
