# Supplementary material for: Tomato-derived carbon quantum dots: composition-driven structure and copper sensing potential
Source: Sci Rep. 2026 Jun 16;16:27639. doi: 10.1038/s41598-026-58128-3 (PMC13538402; doi:10.1038/s41598-026-58128-3)
Supplement: Supplementary file 1 [file 41598_2026_58128_MOESM1_ESM.docx]

**Supplementary file**

**Tomato-Derived Carbon Quantum Dots: Composition-Driven Structure and Copper Sensing Potential**

Jovana Periša^1+^, Jan Hočevar^2+^ ,Bojana Milićević^1^, Jernej Iskra^2^, Boštjan Genorio^2^, Darja Lisjak^3^, Miroslav D. Dramićanin^1^, Jelena Papan Djaniš^1,2*^

^1^ Centre of Excellence for Photoconversion, Vinča Institute of Nuclear Sciences, National Institute of the Republic of Serbia, University of Belgrade, 11351 Belgrade, Serbia

^2^ Faculty of Chemistry and Chemical Technology, University of Ljubljana, 1000 Ljubljana, Slovenia

^3^ Department for the Synthesis of Materials, Jožef Stefan Institute, 1000 Ljubljana, Slovenia

^+^authors equally contributed

**Characterization of Materials**

Infrared (IR) spectra of the synthesized CQD were recorded between 4000 cm^-1^ and 400 cm^-1^ using a Bruker Alpha-II FT-IR spectrophotometer with an ATR module. The number of scans was 20 and the resolution was 4 cm^-1^.

Raman spectra were recorded with Bravo Handheld Raman Spectrometer, Bruker Optic. Raman spectra were recorded across the entire operating range of the instrument (4000-400 cm^-1^) with automatically set recording parameters (integration time and number of scans). If necessary, the number of scans was increased between 5 and 70, while the integration time remains the same as that determined by the automatic Raman spectroscopy method.

Electrokinetic measurements (zeta potential) and dynamic light scattering (DLS) analysis of CQDs dispersed in double-deionized water at a concentration of 1 mg/mL were performed using a Litesizer 500 (Anton Paar). The pH was adjusted with HCl and NaOH solutions (0.1 or 1 M).

For Nuclear magnetic resonance (NMR) measurements, 40 mg of dry, pre-isolated CQD was dissolved in 0.5 mL of deuterated NMR solvent. Deuterium oxide (D₂O) was used as the NMR solvent for analysis of the biomass extract. In contrast, DMSO-d₆ was employed to analyze the reaction intermediate formed between the dopant 3-aminobenzoic acid and the biomass extract. The solution was then filtered and transferred to an NMR tube for analysis. ^1^H, ^13^C and 2D-HSQC NMR spectra were recorded at 25 °C using a Bruker Avance III 600 spectrometer. The following acquisition parameters were used for scanning 1H NMR spectra: number of scans (ns) = 64, dummy scans (ds) = 2, relaxation delay (d1) = 2.00 s, and pulse program = zg30. The following acquisition parameters were used for scanning 13C NMR spectra: number of scans (ns) = 10240, dummy scans (ds) = 4, relaxation delay (d1) = 2.00 s, and pulse program = zgpg30. The following acquisition parameters were used for scanning 2D-HSQC NMR spectra: F2 range = 10 to 0 ppm, F1 range = 158 to 8 ppm, number of scans (ns) = 24, dummy scans (ds) = 16, number of increments (ni) = 256, relaxation delay (d1) = 1.47 s, and pulse program = hsqcetgpsi2. The spectra were processed with Bruker TopSpin 4.1.1 and MestReNova software.

X-ray photoelectron spectroscopy (XPS) measurements were performed using a Versa Probe 3 AD (PHI, Chanhassen, USA) equipped with a monochromatic Al Kα X-ray source. The source operated at an accelerating voltage of 15 kV and an emission current of 13.3 mA. Powder samples were mounted on double-sided Scotch tape and positioned at the center of the XPS holder. Spectra were collected for each sample over a 200 μm by 200 μm analysis area, with the charge neutralizer activated during data acquisition. Survey spectra were recorded with a pass energy of 224 eV and a step size of 0.8 eV. High-resolution (HR) spectra were captured with a pass energy of 27 eV and a step size of 0.05 eV. To obtain high-quality spectral data with a good signal-to-noise ratio, at least 10 sweeps were performed for each measurement. The energy scale and any charging effects in the XPS spectra were corrected by referencing the C=C peak in the C 1s spectrum of the carbon support, with a binding energy (BE) of 284.5 eV. Spectral deconvolution was carried out using MultiPak 9.9.1 and Casa XPS software, with Shirley background correction applied to all spectra.

The morphology of the samples was examined using a transmission electron microscope (TEM, Jeol 2100). The CQDs were drop-cast from aqueous dispersions onto a copper TEM grid with a carbon support and left to dry in air.

Photoluminescence properties (PL) were examined using a spectrofluorometric system (Fluorolog-3 Model FL3-221, Horiba JobinYvon) with a 450 W xenon lamp as the excitation source for steady-state emission measurements. The total volume of each sample was 2 mL. The concentration of the stock solutions of Cu^2+^ ions used in the experiment was 10^−5^ M. The metal-ion stock solutions were added in various volumes to the dispersion of NPs (NPs concentration 1 mg/mL) to adjust the final concentrations measured. Each PL spectrum was recorded 5 minutes after adding the Cu^2+^ ions. The luminescence quantum efficiency (QY) was assessed with the HORIBA Scientific FluoroLog-QM modular fluorometer, equipped with an integrating sphere, using (1,4-bis(5-phenyloxazol-2-yl)benzene) - POPOP as a reference material.

**Fig S1**: DLS analysis in water of a) CQD F, and b) CQD S; Comparison of emission intensity of freshly prepared colloid and colloid stored for 6 months of c) CQD F and d) CQD S; d) Image of colloids after 6 months of storage.

**Fig S2**: Zeta potentials of CQD F and CQD S

**Fig S3**: Survey spectra with quantification of a) CQD F, and b) CQD S.

**Fig S4**: 2D HSQC NMR spectra of extractives obtained from tomato fruit (a) and stem (b) biomass.

**Fig S5**: a) FTIR spectra of a tomato fruit biomass, compared with dopant 3-ABA, extractives from fruit biomass obtained with ultrasound, mixture of exctractives and 3-ABA, and CQD F b) FTIR spectra of a tomato steam biomass, compared with dopant 3-ABA, extractives from fruit biomass obtained with ultrasound, mixture of exctractives and 3-ABA, and CQD S.

**Table S1:** The masses of reagents and products at each stage of CQD synthesis.

| Biomass | m (biomass) [mg] | m (3-ABA) [mg] | m (extractives after 1^st^ stage) - US [mg] | m (extractives after 1^st^ stage) [mg] | m (residue after 2^nd^ stage) [mg] | m (CQD) [mg] |
| --- | --- | --- | --- | --- | --- | --- |
| Tomato fruit | 300 | 300 | 180 | 185 | 414 | 18 |
| Tomato stem | 300 | 300 | 66 | 60 | 313 | 26 |

*US - ultrasound

**Fig S6**: Comparison of ^1^H and ^13^C NMR spectra of tomato fruit (a) and stem (b) biomass extractives obtained with and without ultrasound-assisted extraction.

**Fig S7**: ^1^H, ^13^C, and HSQC NMR spectra of the dopant 3-aminobenzoic acid. recorded in DMSO-d₆ NMR solvent.

**Fig S8**: 2D HSQC NMR spectra of the second stage of the reaction during the synthesis of CQDs derived from a) tomato fruit biomass extractives, and b) steam biomass extractives

**Fig S9**: ^1^H, ^13^C, and 2D HSQC NMR spectra of of the dopant 3-aminobenzoic acid recorded in DMSO-d₆ supplemented with D₂O.

**Fig S10**: Comparison of the aromatic regions in the ^1^H NMR spectra of the dopant, CQD S, and CQD F.

**Fig S11**: Comparison under UV light of 3-ABA CQDs (left) and CQD S (right).
